# Point-of-Care Ultrasound in Blunt Chest Trauma: Diagnostic Performance and Clinical Relevance

**DOI:** 10.7759/cureus.105427

**Published:** 2026-03-18

**Authors:** João Matias, Jaime Miguel Abreu

**Affiliations:** 1 Department of Medical Sciences, Beira Interior University, Covilhã, PRT; 2 Polyvalent Intensive Care Unit, Local Health Unit of Castelo Branco, Castelo Branco, PRT

**Keywords:** blunt injuries, chest trauma, efast, pocus, ultrasound

## Abstract

Chest trauma is a leading cause of death and illness among trauma patients, accounting for a significant proportion of emergency department admissions. Therefore, the early identification of intrathoracic injuries is crucial to ensure effective treatment and reduce the risk of complications, particularly those affecting the heart and lungs. Complementary diagnostic methods, such as ultrasound, due to their non-invasive nature and ability to provide real-time imaging, are therefore essential for making informed decisions based on correctly identifying these complications. In this context, this study reviews the scientific literature on the role of ultrasound in assessing patients with blunt chest trauma. This review aims to emphasise the importance of ultrasound as a complementary diagnostic tool in optimising urgent and emergency care for patients with blunt chest trauma by comparing its advantages and limitations with those of other complementary diagnostic tools for the various blunt trauma consequences, such as pneumothorax, aortic rupture, pericardial effusion, pulmonary contusion, haemothorax and rib fracture.

## Introduction and background

​​​​​​Chest trauma is a significant cause of death and illness in trauma victims, accounting for a considerable proportion of emergency department admissions (10% or more of all trauma admissions) [1,2]. Given that the heart and lungs are located in the thorax, early identification of intrathoracic injuries is essential to ensure effective treatment and reduce complications [2].

This type of trauma can result in several complications, including pneumothorax, haemothorax, rib fractures, aortic rupture, pulmonary contusion and pericardial effusion [3]. Therefore, it is essential to identify these conditions correctly and early in order to make effective therapeutic decisions, which have a direct impact on the patient's final prognosis [4]. Consequently, complementary diagnostic methods are essential [5].

Ultrasound is a non-invasive Complementary Diagnostic and Therapeutic Method (CDTM) that uses ultrasound waves to visualise internal body structures. It is based on the emission of high-frequency waves and the analysis of the echoes reflected by different tissues, allowing real-time imaging [6]. It is naturally essential in patient assessment due to its characteristics, such as rapidity, portability, the absence of ionising radiation and low cost, among others [7].

Point-of-care ultrasound (POCUS) is a very relevant concept in this context. This involves the doctor performing an ultrasound scan at the patient's bedside, allowing for a rapid diagnosis and guiding treatment. It is considered an extension of the physical examination, dynamically integrating into clinical assessment and immediate decision-making. Unlike traditional ultrasound, the doctor performs all image acquisition and interpretation while in contact with the patient, guiding any additional diagnostic and/or therapeutic procedures [5, 8]. This approach enables faster action, particularly in trauma scenarios where time can be critical for the patient's clinical situation [9]. In trauma care, POCUS is integrated into the systematic approach recommended by the Advanced Trauma Life Support (ATLS) protocol, particularly during the primary survey as an adjunct to the assessment of life-threatening injuries. Within this framework, the FAST and eFAST protocols play a central role, enabling the rapid identification of intrathoracic and intra-abdominal complications in unstable patients and supporting early therapeutic decisions [2,3]. This approach enables faster action, particularly in trauma scenarios where time can be critical for the patient’s clinical situation.

Despite its many advantages, ultrasound also has important limitations. It is highly operator-dependent, and its diagnostic accuracy may vary according to the examiner’s experience and technical skill. Image acquisition can also be more difficult in certain situations, such as obesity, subcutaneous emphysema, and poor patient cooperation. In addition, ultrasound does not provide the same comprehensive anatomical detail as computed tomography (CT), particularly for deeper structures or lesions not directly accessible through standard acoustic windows. Therefore, although it is extremely useful in the initial assessment of blunt chest trauma, ultrasound should be interpreted in conjunction with the clinical findings and complemented by other imaging modalities, whenever necessary [7,9].

Chest X-rays and CT scans have become commonplace in the assessment of chest trauma over time. However, ultrasound has become increasingly popular due to its portability and ease of use, making it a valuable CDTM for the early management of chest trauma [10]. Its portability also enables its use in the pre-hospital setting, thereby reducing the time between the detection of trauma complications and therapeutic intervention [7]. Conversely, the Focused Assessment with Sonography for Trauma (FAST) and its extended version (extended Focused Assessment with Sonography for Trauma (eFAST)) protocols enable the early detection of thoracic complications during the initial assessment of trauma patients, thereby improving diagnostic accuracy and reducing the time taken for therapeutic intervention [11]. FAST is a POCUS protocol used in the initial assessment of trauma patients. It is particularly useful in cases of haemodynamic instability and aims to quickly identify the presence of potentially pathological free fluid. It focuses on four ultrasound windows: the pericardial, perihepatic, perisplenic and pelvic regions. Subsequently, eFAST emerged as an evolution of the original FAST protocol, expanding it to include thoracic assessment for the detection of pneumothorax and haemothorax. This increases the usefulness of the examination in the overall approach to chest trauma [11].

Although CT is considered the gold standard for detecting chest trauma, its availability may not always be possible, particularly in trauma scenarios where action is often required in the pre-hospital setting. Therefore, it may not be feasible to use CT scans in unstable patients [4].

This review examines the use of ultrasound in the assessment of patients with blunt chest trauma and its various complications. It outlines the advantages and limitations of ultrasound compared with other CDTMs. For each trauma-related complication, a comparison is made with the *gold standard* and/or the approach used in the initial assessment of the condition. Similarly, the use of ultrasound in different types of chest injury caused directly or indirectly by trauma is analysed based on the scientific literature consulted. The aim is to understand the importance of this emerging CDTM in improving urgent and emergency care for patients with blunt chest trauma.

## Review

Methodology

This paper was written as a structured narrative review. A literature search was performed using the PubMed bibliographic database. Searches were conducted between January 2025 and February 2026, corresponding to the period during which the literature search was performed, updated and refined for the preparation of this paper.

The search strategy included the keywords "ultrasound" and "thoracic trauma". Broad search terms were deliberately selected to avoid overly narrowing the results and minimise the risk of excluding potentially relevant studies. To improve the sensitivity of the search strategy, synonyms and related terms were also considered. In addition to the main keywords, alternative expressions such as “blunt thoracic trauma”, “chest injury”, “thoracic injury”, “ultrasonography”, “sonography”, “POCUS”, and “eFAST” were used when appropriate, to reduce the risk of missing relevant literature. The search was further limited to open-access articles published in Portuguese, English, Spanish and French.

The initial search identified approximately 237 articles. After screening the titles, 188 articles were excluded for not meeting the inclusion criteria. The inclusion criteria comprised articles addressing the use of ultrasound in blunt chest trauma, particularly in the diagnosis of thoracic injuries and trauma-related complications, as well as studies allowing comparison with other imaging modalities when relevant. Articles not related to the topic, focused on non-thoracic trauma, penetrating trauma or clearly unrelated objectives were excluded at this stage. This left 49 articles for further assessment. Of these, a further 20 were excluded after reviewing the abstracts, because they were not sufficiently relevant to the objective of the review, leaving 29 articles that were read in full. An additional targeted search was then conducted, focusing particularly on other complementary diagnostic modalities used in evaluating the pathologies addressed, to establish comparisons with ultrasound. This additional search identified three further relevant articles. In total, 32 articles were reviewed in full.

It is important to note that this review only considered blunt thoracic injuries. Penetrating lesions were excluded from this review because blunt and penetrating trauma involve different mechanisms of injury, diagnostic approaches, and management strategies. The review focused specifically on blunt chest trauma, in which ultrasound may be particularly useful for the early identification of occult intrathoracic injuries that are not immediately evident on physical examination.

The main characteristics of the studies included in this review are summarised in Table 1.

**Table 1:** Summary of the studies included in this review. POCUS, point-of-care ultrasound; ARDS, acute respiratory distress syndrome; FAST, Focused Assessment with Sonography for Trauma; eFAST, extended Focused Assessment with Sonography for Trauma

Author	Year	Study type	Main focus
Tian et al. [1]	2023	Meta-analysis	Emergency chest ultrasound in traumatic pneumothorax
Dennis et al. [2]	2017	Review	Thoracic trauma
Hyacinthe et al. [3]	2012	Prospective study	Ultrasound diagnosis of thoracic trauma injuries
Fathi et al. [4]	2024	Systematic review and meta-analysis	Whole-body CT in trauma assessment
Whitson and Mayo [5]	2016	Review	Ultrasonography in emergency medicine
Gavrilă et al. [6]	2020	Case report and literature review	Ultrasound as first imaging method in lung disease
Choi et al. [7]	2020	Clinical guideline	Guidance for POCUS in emergency care
Smallwood and Dachsel [8]	2018	Commentary/review	Evidence and clinical value of POCUS
Irwin and Cook [9]	2016	Review	Advances in thoracic POCUS
Feletti et al. [10]	2018	Review	Chest ultrasonography in extreme environments
Montoya et al. [11]	2016	Review	Evolution of FAST and eFAST trauma ultrasound protocols
Griffard and Kodadek [12]	2024	Review	Management of blunt chest trauma
Rovida et al. [13]	2022	Clinical review	Lung ultrasound in blunt chest trauma
Chan et al. [14]	2020	Systematic review (Cochrane)	Ultrasound vs. chest radiography for pneumothorax
Staub et al. [15]	2018	Systematic review and meta-analysis	Ultrasound diagnosis of traumatic pneumothorax and haemothorax
Sheng et al. [16]	2024	Meta-analysis	Diagnostic performance of ultrasound vs. radiography in pneumothorax
Mouawad et al. [17]	2020	Review	Blunt thoracic aortic injury management
Mokrane et al. [18]	2015	Review	Imaging diagnosis of thoracic aortic trauma
Liu and Huang [19]	2018	Review	Ultrasound in aortic dissection management
Habib et al. [20]	2023	Review	Descending thoracic aortic emergencies
Hoit [21]	2023	Review	Pathophysiology and management of pericardial effusion
Alerhand et al. [22]	2022	Review	Pericardial tamponade in emergency medicine
Alerhand & Carter [23]	2019	Review	Echocardiographic findings suggesting cardiac tamponade
Offenbacher et al. [24]	2021	Case series	Traumatic haemopericardium and cardiac tamponade
Yıldız Potter et al. [25]	2023	Experimental study	Automated ultrasound detection of pericardial effusion
Van Diepen et al. [26]	2024	Systematic review	CT classification of pulmonary contusion
Abbasi et al. [27]	2018	Diagnostic accuracy study	Ultrasound B-lines in lung contusion
Hosseini et al. [28]	2015	Systematic review and meta-analysis	Ultrasound vs. radiography for pulmonary contusion
Sayed et al. [29]	2022	Observational study	Lung ultrasound score predicting ARDS after trauma
Abboud and Kendall [30]	2003	Observational study	Emergency ultrasound diagnosis of haemothorax
Çelik et al. [31]	2021	Diagnostic accuracy study	Ultrasound detection of rib fractures
Gilbertson et al. [32]	2022	Systematic review and meta-analysis	Ultrasound diagnosis of rib fractures

Problems caused by blunt chest trauma

Blunt chest trauma typically occurs when external force is suddenly applied to the chest without penetrating the chest wall. Road accidents are the main cause of trauma, followed by falls and direct impacts during physical activity [2,12]. The transmitted energy can cause direct compression or inertial forces associated with high-energy mechanisms, which can affect intrathoracic structures such as the lungs, large blood vessels (e.g. aorta), ribs and heart.

This section addresses the most common complications of this type of trauma, which include pneumothorax, haemothorax, rib fractures, aortic rupture (although this is relatively uncommon, it is addressed due to its severity and consequent significant threat to life), pulmonary contusion, and pericardial effusion. These complications rarely occur in isolation, and it is common for several to coexist in the same polytrauma patient [12]. This emphasises the importance of a systematic and guided diagnostic approach from the initial stages of care.

Pneumothorax

Pneumothorax is characterised by the presence of air in the pleural space, which can lead to partial or complete lung collapse and impaired respiratory function [13]. It is a complication that should be considered in the context of trauma, as it is one of the most common causes of potentially fatal thoracic complications. Early diagnosis is therefore crucial, as progression can lead to haemodynamic instability and a life-threatening condition known as tension pneumothorax. This occurs when a progressive accumulation of air exists in the pleural space. This increases intrathoracic pressure, compressing the affected lung increasingly, deviating the mediastinum and compromising venous return to the heart, thereby leading to reduced cardiac output. This situation is a medical emergency requiring decompression of the pleural space to prevent cardiovascular collapse and respiratory failure [11].

Thoracic ultrasound has become an essential diagnostic tool for traumatic pneumothorax, particularly in emergencies. This imaging technique allows for a rapid assessment, often in a pre-hospital setting by emergency medical teams at the scene of an incident or while transporting a patient. This application, therefore fits the concept of POCUS [11,13].

The most relevant ultrasound signs for diagnosing pneumothorax have been studied and are now firmly established in clinical practice. These signs are absence of lung sliding, absence of B lines, presence of lung point, and absence of lung pulse [14].

Lung sliding refers to the movement of the visceral pleura over the parietal pleura during breathing. Under normal circumstances, this movement is visible as a hyperechoic line that oscillates during inspiration and expiration. Its absence suggests separation between the pleurae, which occurs when there is air in the pleural space, as in pneumothorax [14,15].

Another important piece of information is the absence of B lines. These vertical hyperechoic lines originate from the pleura and extend downwards within the ultrasound image, oscillating with respiration. Their presence normally indicates contact between the pleurae, so their absence reinforces the suspicion of pneumothorax [14,15].

The presence of the lung point is an extremely specific sign of pneumothorax. It marks the point at which the visceral pleura begins to separate from the parietal pleura of the chest wall, at the edge of the pneumothorax. Identifying this point confirms the diagnosis by excluding other potential causes of the absence of sliding, such as pleural adhesions [14,15].

The lung pulse corresponds to the rhythmic oscillation of the pleural line caused by the heartbeat. Its presence indicates contact between the pleurae, while its absence suggests pleural separation, which may be associated with pneumothorax [14,15].

Finally, M mode (motion mode) provides a temporal representation of pleural movement. Under normal conditions, this mode displays the seashore sign, characterised by a stable surface layer (the soft tissues of the chest wall) contrasting with a granular-looking deeper area corresponding to pleural movement. In pneumothorax, where there is no sliding, this pattern disappears and is replaced by the barcode sign, characterised by parallel horizontal lines indicating an absence of movement [14,15]. Thus, these signs, especially when combined, make ultrasound a very useful tool for diagnosing pneumothorax [13].

CT is still considered the gold standard for diagnosing pneumothorax, particularly in cases where there is doubt or when it is necessary to accurately assess the extent of the injury. However, CT may not be feasible in unstable trauma patients [15]. In such cases, chest ultrasound plays a pivotal role by enabling a rapid and safe assessment at the patient's bedside, particularly when used in conjunction with the eFAST protocol.

Furthermore, chest ultrasonography has performance characteristics like those of chest CT, and the addition of chest ultrasound improves diagnostic accuracy and efficiency [5].

Ultrasound demonstrates diagnostic superiority compared to supine chest radiography, which is traditionally used in the initial approach to polytrauma patients. Several studies have shown that radiography can be ineffective in up to 50% of cases of pneumothorax, particularly in the case of small and anterior pneumothoraxes [16]. A Cochrane meta-analysis concluded that ultrasound has significantly higher sensitivity than radiography (91% vs. 47%), with similar specificity [14]. Therefore, when CT is unavailable or inappropriate, ultrasound is the most effective bedside test for swiftly ruling out or confirming pneumothorax.

As discussed before, eFAST has been shown to be more effective than conventional chest radiography at detecting pneumothorax, while retaining the advantages of portability and the absence of ionising radiation. It is currently considered an essential tool in the initial assessment of polytrauma patients [11]. However, ultrasound has limitations, despite its numerous advantages. The presence of subcutaneous emphysema, obesity or open chest wall wounds can make visualising the pleural structures difficult. Furthermore, the success of the technique depends on the operator's experience and the environment in which it is performed. In trauma scenarios, where urgent transport to hospital is required due to other life-threatening complications, it can be challenging due to a lack of stability, particularly due to oscillations resulting from transport [13].

In summary, chest ultrasound is a highly effective, rapid and safe method of diagnosing traumatic pneumothorax. It is particularly useful when more complex imaging tests cannot be performed, such as in the treatment of patients in a pre-hospital setting, enabling the necessary procedures to be initiated quickly.

Rupture of the aorta

Traumatic rupture of the thoracic aorta is one of the leading causes of death in cases of closed/blunt trauma, surpassed only by head injuries. According to autopsy results, 80% of patients who suffer aortic rupture die at the scene of the accident, and 46% ultimately die among those who survive and are hospitalised [17,18].

The aortic isthmus, located between the left subclavian artery and the arterial ligament, is the most common site of rupture due to its anatomical vulnerability at the transition between the fixed and mobile aorta [18]. The mechanism of injury is usually associated with high-energy trauma involving sudden deceleration, such as traffic accidents or falls from a great height. Three biomechanical processes have been described: shear and stretching forces; compression of the aorta between the sternum and spine; and the intrinsic fragility of the isthmus [17]. In clinical terms, the presentation is somewhat non-specific and vague, often masked by other concomitant injuries. Chest pain, dyspnoea and shock are often present, but diagnosis is essentially based on imaging [18].

Although chest radiography is usually the first examination performed on polytrauma patients, it has limited sensitivity. Although there are suggestive findings, such as mediastinal widening, aortic arch blurring, tracheal deviation or left haemothorax, these do not enable aortic rupture to be either excluded or confirmed [17].

When used in accordance with the eFAST protocol, POCUS has a somewhat limited role in the direct assessment of the aorta. However, as outlined in other sections of this review, it can detect indirect complications, such as haemothorax or pericardial effusion resulting from rupture [17]. On the other hand, transoesophageal echocardiography (TEE) is more effective at identifying intimal flaps, which are lacerations of the innermost layer of an artery, as well as periaortic haematomas. It is particularly useful in unstable patients who cannot be transported to radiology [19]. This examination enables direct visualisation of the aortic intima and the early detection of dissections or incipient ruptures with high sensitivity and specificity. Additionally, it enables real-time evaluation of the extent of dissections and haemodynamic repercussions by detecting pericardial effusion or signs of cardiac tamponade. Furthermore, it spares haemodynamically unstable patients from being transported to the CT room, thereby reducing the risk of deterioration [19].

However, despite its usefulness, TEE is an operator-dependent examination and may have limitations in assessing more distal segments of the thoracic aorta or abdominal aorta, often being complemented by CT angiography once the patient has stabilised. Another limitation is that it cannot be used in a pre-hospital setting, which would be useful in a condition that has such a high mortality rate at the site of occurrence [19].

Contrast-enhanced CT (CT angiography) is currently considered the real gold standard for the diagnosis of traumatic thoracic aortic rupture in stable patients [18], allowing the identification of intimal flap, pseudoaneurysm, mediastinal haematoma and contrast extravasation, as well as enabling the classification of the injury [18].

The development of multi-slice systems has enabled submillimetre cuts and rapid acquisition, thereby optimising the sensitivity and specificity of examinations [20]. Conventional angiography, once considered the gold standard, is now only used in specific cases, particularly when endovascular intervention is required [18].

In summary, traumatic rupture of the thoracic aorta is a rare but highly lethal condition whose diagnosis essentially depends on imaging. While radiography and ultrasound only provide indirect evidence, TEE can be useful in unstable patients. Nevertheless, CT angiography remains the preferred method and the real gold standard, enabling a rapid, accurate and comprehensive evaluation of the injury.

Pericardial effusion

Pericardial effusion is a serious complication of chest trauma. As the pericardial cavity is filled with plasma ultrafiltrate, a pericardial effusion is only considered present when more than 50 mL of fluid accumulates between the two serous layers of the pericardium [21]. This condition is particularly relevant as it can progress to cardiac tamponade, which is life-threatening. Cardiac tamponade corresponds to haemodynamic instability resulting directly from pericardial effusion. This occurs when the intrapericardial pressure exerted by the fluid exceeds the intracardiac pressure, which impairs the filling of the heart chambers, primarily the right one [22,23]. It is less common in cases of blunt trauma than penetrating trauma, and is usually caused by cardiac rupture, injury to a large vessel, or injury to the bone structure [24].

Clinically, the presentation is often non-specific. Although symptoms such as dyspnoea, chest pain, fatigue and haemodynamic instability may be present, Beck's classic triad (hypotension, jugular venous distension and muffled heart sounds), which is pathognomonic, is not very sensitive and occurs in less than one-third of patients [21,22]. Therefore, diagnosis is primarily based on imaging methods. 

Ultrasound when into the eFAST protocol is considered the primary examination for blunt chest trauma. Through the subxiphoid window, or alternatively the parasternal or apical window, anechoic or hypoechoic fluid can be identified between the epicardium and the pericardium. This allows for the diagnosis of traumatic pericardial effusion [21]. In addition to identifying fluid, ultrasound can detect signs of haemodynamic repercussions indicative of cardiac tamponade. These signs include diastolic collapse of the right ventricle (a finding with high specificity of around 75%-90%, but low sensitivity of 48%-60%; it is more specific than right atrial collapse, but less sensitive), systolic collapse of the right atrium (earlier specificity of 33%-100%), dilation of the inferior vena cava with reduced compressibility, and marked respiratory variation in mitral and tricuspid flows, suggesting pulsus paradoxus (corresponding to a drop in systolic blood pressure of more than 10 mmHg during inspiration) [23].

These findings demonstrate that ultrasound is not only a diagnostic tool, but also enables the severity to be characterised, allowing early signs of tamponade to be recognised and facilitating immediate decision-making. The quickness, availability, and applicability of ultrasound at the bedside of an unstable patient make it the ideal method in emergencies, surpassing more time-consuming alternatives such as CT [21]. However, despite its high utility, ultrasound has limitations: the results may depend on the person performing the scan; the procedure may be hindered by limited acoustic windows (e.g. obesity or subcutaneous emphysema or air); and ultrasound has limited tissue characterisation capabilities [21].

According to the European Society of Cardiology (ESC) recommendations, CT is considered a second-line examination when ultrasound is inconclusive or when more detailed characterisation of the effusion is desired. CT enables more precise quantification and localisation of pericardial fluid, identification of complex or loculated effusions, and differentiation between effusion, clot, or epicardial fat. It can also exclude concomitant pleural effusion. However, it has limitations compared to cardiac ultrasound, for instance, the patient must hold their breath, which may be impractical for polytraumatised patients [21].

Technological advances also make it possible to use artificial intelligence algorithms applied to POCUS, such as YoloV3. An experimental study has shown that this can detect and localise pericardial effusion with high sensitivity (89%) and specificity (92%), which could be an added asset in trauma settings in the future, especially where ultrasound experience is limited [25].

In conclusion, despite its limitations, ultrasound is essential for detecting pericardial effusion, with CT reserved for situations requiring further investigation.

Pulmonary contusion

Pulmonary contusion is a parenchymal injury that is common in cases of blunt chest trauma, occurring in 30% to 75% of such cases. It is caused by intralveolar and interstitial haemorrhage secondary to capillary rupture resulting from compression and shear forces, without obvious laceration of the parenchyma, which impairs gas exchange [26]. It can also cause alveolar collapse, consolidation and atelectasis. This injury is clinically significant as it is associated with potential complications such as pneumonia, respiratory failure and acute respiratory distress syndrome (ARDS), with a mortality rate of 10%-25% [27].

In recent years, chest ultrasound has become an important tool for diagnosing pulmonary contusion. As early as 2015, a meta-analysis demonstrated a high level of sensitivity (94%) and specificity (89%) in the detection of pulmonary contusion [28]. The most characteristic ultrasound signs are the presence of multiple diffuse B-lines (more than six per field), which have a sensitivity of 90% and a specificity of 94%, and the identification of hypoechoic peripheral parenchymal lesions (C-lines), which have a specificity of 100% but a sensitivity of 70%. Prospective studies have also shown that the extent of contusion quantified by ultrasound using the Lung Ultrasound Score (LUS) correlates strongly with CT (with a correlation coefficient of 0.78), making ultrasound a highly reliable CDTM for this pathology. An LUS value greater than 4 at the time of admission allows the occurrence of ARDS to be anticipated within the first 72 hours post-trauma (sensitivity 91.7%; specificity 84.2%) [29], enabling preventive action to be taken. In addition to the initial diagnosis, chest ultrasound is a very useful tool for monitoring these patients over time, as it allows the extent of the contusion to be monitored dynamically without the need to transport critically ill patients, which is particularly beneficial in pre-hospital trauma scenarios [29].

Diagnostic imaging plays a key role. Chest CT is considered the gold standard for detecting the extent of parenchymal involvement from the contusion, with high sensitivity. The most used classification methods are based on the percentage of lung volume affected. Values of 18%-24% or more have been shown to be associated with longer stays in hospital and in intensive care, a higher incidence of pneumonia and ARDS, and a greater need for mechanical ventilation [26]. Chest X-rays, on the other hand, have reduced sensitivity (44%) and can take up to 48 hours to reveal contusion signs, although they have high specificity (98%) [28]. Consequently, 73% of pulmonary contusions are only visible on CT scans compared to chest X-rays. Therefore, integrating chest ultrasound into the approach to patients with blunt chest trauma enables not only the correct and rapid diagnosis of pulmonary contusion, but also its classification and monitoring, thus complementing traditional imaging methods.

Haemothorax

Haemothorax is a possible complication of blunt chest trauma, affecting around one-third of patients with chest injuries. It is characterised by the accumulation of blood between the visceral and parietal pleura, which is typically caused by injury to the intercostal, pulmonary or large vessels. This complication is clinically significant as it can lead to lung collapse, haemorrhagic shock, and complications such as retained haemothorax and empyema [15].

Chest X-rays are commonly used as an initial approach. However, they are not very effective at detecting haemothorax, particularly in patients in the supine position. In this position, approximately 175 mL of blood is needed for it to be visible. In the upright position, only 50-100 mL is needed. For this reason, CT is considered the gold standard: not only is it highly sensitive, but it also allows the volume of blood to be quantified and associated complications, including organised clots, to be identified [15].

As in other previous pathologies, chest ultrasound has progressed in recent years as a first-line CDTM in trauma, integrating the eFAST protocol. This examination enables the identification of small volumes (from around 20 mL). These are detected by the presence of an anechoic or hypoechoic area between the pleura and the diaphragm, mainly in the costophrenic angle. This area may contain internal echoes or mobile clots, which help distinguish it from a simple pleural effusion. This is a major advantage over CT, which does not allow for this distinction. A 2018 meta-analysis reported an overall sensitivity of 60% and a specificity of 98%, with an area under the curve (AUC) of 0.953 for diagnosing haemothorax. This makes it a highly accurate diagnostic method [15].

A study conducted in 2003 at Denver Health Medical Centre evaluated 142 patients with blunt chest trauma, comparing ultrasound performed in the emergency department with CT scans. Sixteen cases of haemothorax were documented, of which only two were identified by ultrasound. The authors reported a sensitivity of 12.5% and a specificity of 98.4% and concluded that it is not very sensitive in detecting small volume haemothorax, although ultrasound is very reliable when positive [30]. However, it is important to note that not all small effusions detected on CT led to clinically significant consequences. Nevertheless, previous studies cited by the same authors had reported sensitivities ranging from 67% to 100% and specificities greater than 99%. This discrepancy may be due to differences in the comparative method used and/or the experience of the operators. It should also be noted that this is an old study and therefore does not reflect the advances in ultrasound technology that have occurred in the last 20 years.

In conclusion, chest ultrasound should be considered an important method of diagnostic imaging in the diagnosis of traumatic haemothorax. It is quick and easy to perform, does not use radiation and is particularly useful for unstable patients or in a pre-hospital setting, as previously discussed. However, careful analysis is required when small volumes are suspected, in which case CT remains the preferred CDTM.

Rib fractures

Rib fractures are the most common thoracic injury in cases of blunt chest trauma. They are often associated with pain and hypoventilation, as well as an increased risk of complications such as pneumonia, pneumothorax or haemothorax [12, 31]. Therefore, it is important to correctly diagnose fractures, since even the presence of a single fracture can influence therapeutic decisions relating to analgesia and the need for hospital surveillance [32].

As with other pathologies, radiography is often the first examination performed on a polytrauma patient in a trauma scenario. However, it has limited sensitivity, especially in non-displaced fractures or those located in difficult-to-visualise areas, which can lead to underdiagnosis. Up to 50% of fractures may go undiagnosed [12]. CT scanning is considered the gold standard, as it enables the number and extent of fractures to be determined and hidden fractures to be detected. However, its use is limited, as mentioned earlier in this review [31,32].

Ultrasound scanning of the thorax has become an increasingly popular method of diagnosing rib fractures, as it is more sensitive than X-rays in certain scenarios, as discussed in other sections [13]. The most characteristic ultrasound findings include discontinuity of the bone cortex, the main diagnostic criterion, and the presence of an adjacent subperiosteal haematoma, an indirect sign, which is often associated with the site of pain reported by the patient [13,32]. It is also important to note that ultrasound has shown greater sensitivity when directed at the site of pain reported by the patient [31]. Furthermore, in prospective studies on chest trauma, ultrasound has demonstrated superior overall performance to X-ray, particularly in detecting lesions that are not visible on X-ray [3].

In a study conducted in an urgency service, ultrasound performed by its medical team was directly compared with CT scanning. Ultrasound had a sensitivity of 91.2% and a specificity of 72.7% for detecting at least one rib fracture, demonstrating good overall diagnostic capability [31]. These results are consistent with those of a systematic review which concluded that chest ultrasound had a sensitivity of 89.3% and a specificity of 98.4% in diagnosing rib fractures [32]. However, ultrasound remains highly operator-dependent and less practical in cases of multiple fractures or polytrauma with diffuse pain, where assessment is more challenging. Nevertheless, ultrasound is highly useful as it allows for more precise analgesia by identifying which ribs are involved and provides prognostic information, since the number of fractures is generally associated with an increased risk of respiratory complications and even mortality [13].

Thus, ultrasound should be considered an important complementary tool in assessing rib fractures in cases of blunt chest trauma. Ultrasound is more accurate than radiography for this purpose, although CT may be used subsequently to better characterise the injuries.

Finally, some limitations of this review should be acknowledged. The literature search was restricted to a single database and only open-access articles were included. Future studies should therefore incorporate multiple databases and a broader range of literature, as well as further evaluate the impact of ultrasound on clinical decision-making and patient outcomes, particularly in pre-hospital settings and unstable patients.

## Conclusions

This review compared the role of ultrasound with other diagnostic methods in the assessment of patients with blunt chest trauma and its associated complications. Overall, thoracic ultrasound is a rapid, safe and highly effective method for diagnosing traumatic pneumothorax. However, ultrasound provides only indirect signs of traumatic rupture of the thoracic aorta, whereas CT angiography allows for a rapid, accurate and comprehensive assessment of this injury. Ultrasound also plays an essential role in the detection of pericardial effusion, with CT being reserved for situations requiring further investigation. In cases of pulmonary contusion, the integration of thoracic ultrasound enables rapid diagnosis and monitoring of disease progression, complementing traditional imaging methods. Furthermore, ultrasound should be considered in the diagnosis of traumatic haemothorax, although careful evaluation is required when small volumes are suspected, in which case CT remains the preferred method. Ultrasound is also useful in the assessment of rib fractures following blunt chest trauma, as it may be more sensitive than conventional radiography.

The importance of ultrasound training for non-radiologist operators should also be emphasised, particularly for those involved in the initial assessment of polytrauma patients. As POCUS is highly operator-dependent, proper training and education are essential to ensure accurate image acquisition and interpretation, thereby improving diagnostic reliability. In addition, the wider implementation of portable ultrasound devices in pre-hospital settings could facilitate earlier diagnosis and more timely clinical decision-making. 

## References

[1] Tian H, Zhang T, Zhou Y, Rastogi S, Choudhury R, Iqbal J (2023). Role of emergency chest ultrasound in traumatic pneumothorax. An updated meta-analysis. Med Ultrason.

[2] Dennis BM, Bellister SA, Guillamondegui OD (2017). Thoracic trauma. Surg Clin North Am.

[3] Hyacinthe AC, Broux C, Francony G (2012). Diagnostic accuracy of ultrasonography in the acute assessment of common thoracic lesions after trauma. Chest.

[4] Fathi M, Mirjafari A, Yaghoobpoor S (2024). Diagnostic utility of whole-body computed tomography/pan-scan in trauma: a systematic review and meta-analysis study. Emerg Radiol.

[5] Whitson MR, Mayo PH (2016). Ultrasonography in the emergency department. Crit Care.

[6] Gavrila IL, Badea RI, Jude C, Socaciu MA, Comsa M, Badea AF (2020). Ultrasound as the first imaging method in severe lung disease. Considerations about a case of pulmonary tuberculosis and review of the literature. Med Ultrason.

[7] Choi WJ, Ha YR, Oh JH (2020). Clinical guidance for point-of-care ultrasound in the emergency and critical care areas after implementing insurance coverage in Korea. J Korean Med Sci.

[8] Smallwood N, Dachsel M (2018). Point-of-care ultrasound (POCUS): unnecessary gadgetry or evidence-based medicine?. Clin Med.

[9] Irwin Z, Cook JO (2016). Advances in point-of-care thoracic ultrasound. Emerg Med Clin North Am.

[10] Feletti F, Mucci V, Aliverti A (2018). Chest ultrasonography in modern day extreme settings: from military setting and natural disasters to space flights and extreme sports. Can Respir J.

[11] Montoya J, Stawicki SP, Evans DC, Bahner DP, Sparks S, Sharpe RP, Cipolla J (2016). From FAST to E-FAST: an overview of the evolution of ultrasound-based traumatic injury assessment. Eur J Trauma Emerg Surg.

[12] Griffard J, Kodadek LM (2024). Management of blunt chest trauma. Surg Clin North Am.

[13] Rovida S, Orso D, Naeem S, Vetrugno L, Volpicelli G (2022). Lung ultrasound in blunt chest trauma: a clinical review. Ultrasound.

[14] Chan KK, Joo DA, McRae AD, Takwoingi Y, Premji ZA, Lang E, Wakai A (2020). Chest ultrasonography versus supine chest radiography for diagnosis of pneumothorax in trauma patients in the emergency department. Cochrane Database Syst Rev.

[15] Staub LJ, Biscaro RR, Kaszubowski E, Maurici R (2018). Chest ultrasonography for the emergency diagnosis of traumatic pneumothorax and haemothorax: a systematic review and meta-analysis. Injury.

[16] Sheng B, Tao L, Zhong C, Gao L (2025). Comparing the diagnostic performance of lung ultrasonography and chest radiography for detecting pneumothorax in patients with trauma: a meta-analysis. Respiration.

[17] Mouawad NJ, Paulisin J, Hofmeister S, Thomas MB (2020). Blunt thoracic aortic injury - concepts and management. J Cardiothorac Surg.

[18] Mokrane FZ, Revel-Mouroz P, Saint Lebes B, Rousseau H (2015). Traumatic injuries of the thoracic aorta: the role of imaging in diagnosis and treatment. Diagn Interv Imaging.

[19] Liu F, Huang L (2018). Usefulness of ultrasound in the management of aortic dissection. Rev Cardiovasc Med.

[20] Habib M, Lindström D, Lilly JB (2023). Descending thoracic aortic emergencies: past, present, and future. Semin Vasc Surg.

[21] Hoit BD (2023). Pericardial effusion and cardiac tamponade pathophysiology and new approaches to treatment. Curr Cardiol Rep.

[22] Alerhand S, Adrian RJ, Long B, Avila J (2022). Pericardial tamponade: a comprehensive emergency medicine and echocardiography review. Am J Emerg Med.

[23] Alerhand S, Carter JM (2019). What echocardiographic findings suggest a pericardial effusion is causing tamponade?. Am J Emerg Med.

[24] Offenbacher J, Liu R, Venitelli Z, Martin D, Fogel K, Nguyen V, Kim PK (2021). Hemopericardium and cardiac tamponade after Blunt thoracic trauma: a case series and the essential role of cardiac ultrasound. J Emerg Med.

[25] Yıldız Potter İ, Leo MM, Vaziri A, Feldman JA (2023). Automated detection and localization of pericardial effusion from point-of-care cardiac ultrasound examination. Med Biol Eng Comput.

[26] Van Diepen MR, Wijffels MM, Verhofstad MH, Van Lieshout EM (2024). Classification methods of pulmonary contusion based on chest CT and the association with in-hospital outcomes: a systematic review of literature. Eur J Trauma Emerg Surg.

[27] Abbasi S, Shaker H, Zareiee F (2018). Screening performance of ultrasonographic B-lines in detection of lung contusion following blunt trauma; a diagnostic accuracy study. Compr J Emerg Med.

[28] Hosseini M, Ghelichkhani P, Baikpour M (2015). Diagnostic Accuracy of Ultrasonography and Radiography in Detection of Pulmonary Contusion; a Systematic Review and Meta-Analysis, Vol. 3.

[29] Sayed MS, Elmeslmany KA, Elsawy AS, Mohamed NA (2022). The validity of quantifying pulmonary contusion extent by lung ultrasound score for predicting ARDS in blunt thoracic trauma. Crit Care Res Pract.

[30] Abboud PAC, Kendall J (2003). Emergency department ultrasound for hemothorax after blunt traumatic injury. J Emerg Med.

[31] Çelik A, Akoglu H, Omercikoglu S (2021). The diagnostic accuracy of ultrasonography for the diagnosis of rib fractures in patients presenting to emergency department with blunt chest trauma. J Emerg Med.

[32] Gilbertson J, Pageau P, Ritcey B, Cheng W, Burwash-Brennan T, Perry JJ, Woo MY (2022). Test characteristics of chest ultrasonography for rib fractures following blunt chest trauma: a systematic review and meta-analysis. Ann Emerg Med.

